# The endoscopic treatment of fourth ventricle outlet obstruction: Report of two children and systematic review

**DOI:** 10.1002/ccr3.8234

**Published:** 2023-11-28

**Authors:** Ali Mulhem, Abdul Masih Alsulaiman, Stefanie Hammersen, Sven Kantelhardt

**Affiliations:** ^1^ Department of Neurosurgery Vivantes Klinikum im Friedrichshain Berlin Germany; ^2^ DPhil Program in Evidence‐Based Health Care, Department of Continuing Education University of Oxford Oxford UK

**Keywords:** endoscopic third ventriculostomy, fourth ventricle outlet obstruction, hydrocephalus, systematic review

## Abstract

Fourth ventricle outlet obstruction (FVOO) is a rare cause of hydrocephalus. In the last century, the standard treatment was the suboccipital craniotomy with magendieplasty or ventriculoperitoneal shunt (VP shunt). Since the beginning of the 21st century, the endoscopic third ventriculostomy (ETV) has been considered a less invasive alternative. The medical literature lacks sufficient reports of FVOO cases and strong evidence about ETV's efficacy in treating this condition. We report two cases of FVOO treated with ETV and review published similar cases. Clinical and radiological findings of two FVOO cases with outcomes after ETV were presented. Moreover, we conducted a systematic review after protocol registration in PROSPERO (CRD42021281474). MEDLINE, Embase, Scopus, and Web of Science were searched from inception till December 31, 2022. Studies were included if they reported cases of FVOO treated initially with ETV. Cases with Chiari malformation, Dandy‐Walker malformation, tuberous sclerosis, trapped fourth ventricle, or space‐occupying lesions were excluded. Two reviewers independently examined title/abstract records in the first stage and full‐text publications in the second for eligibility. The primary outcome was the recurrence rate, defined by the need for re‐ETV or other invasive treatments (e.g., VP shunt or magendieplasty). Other outcomes included clinical state at follow‐up and mortality. Two cases, a 3‐year‐old male and 3.5‐year‐old female, with FVOO, were treated with ETV in our department by the same neurosurgeon (SH) in 2013 and 2021. Both cases improved significantly after ETV, and there was no recurrence through the follow‐up. Besides the present cases, we found 57 other cases of FVOO treated with ETV reported in 17 studies between 2001 and 2021. The median age was 26 years, with an IQR from 2.4 to 59 years, and 56% of cases were females. The recurrence rate was 32% in the sample (19 out of 59), with a 95% CI from 21% to 46%. The median time to recure was 2 months with IQR from 1.25 to 26. A VP shunt was the treatment for recurrence in 68% and a re‐ETV in 32%. At the follow‐up (41 ± 29 months), only one case died, and one deteriorated clinically. FVOO is a rare cause of hydrocephalus encountered mainly in the first or sixth decades of life. ETV provides the first reasonable treatment. Despite the moderate recurrence rate, the outcomes are favorable.

## Graphical abstract

Hydrocephalus with dilatation of all ventricles due to FVOO with a displacement of the brainstem (red arrow) and the cerebellum (blue arrow).

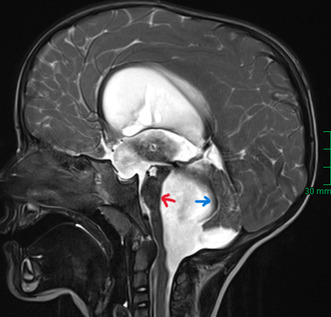

## 1 BACKGROUND

Fourth ventricle outlet obstruction (FVOO) is a rare cause of hydrocephalus. This condition includes obstruction of the foramen (Magendie and Luschka) in the floor of the fourth ventricle without aqueductal stenosis. The resulting hydrocephalus is designated by widening the four compartments of the ventricular system (hydrocephalus internus).^1^, ^2^ FVOO could be classified as a primary condition resulting from a congenital membrane in the fourth ventricle, after intraventricular bleeding (especially in premature newborns), after infection, or from idiopathic etiology, or it could be classified as secondary to other primary lesions such as Chiari malformation or tumors, etc.^3^ Here, we are restricting our study and discussion on the primary variation of this condition.

Although primary FVOO was first described in the late 19th century by Wernicke and Macewen,^4^, ^5^ the medical literature still lacks sufficient studies of this rare condition and its best treatment modality. In the 20th century, neurosurgeons undertook suboccipital craniotomy with magendieplasty or ventriculoperitoneal shunt (VP shunt) implantation as standard treatments^1^, ^6^; however, these approaches are invasive and accompanied by high postoperative or lifelong complication rates. Since the hydrocephalus caused by FVOO is from the occlusive type, VP shunting was frequently applied as a standard treatment, especially in pediatric patients.^3^ More recent recommendations favor endoscopic third ventriculostomy (ETV) as a minimally invasive alternative treatment. Since the beginning of the 21st century, ETV has been considered a possible treatment of FVOO.^1^, ^2^, ^6^ Because of the rarity of this condition, there is insufficient evidence about the efficacy of ETV in treating it, especially regarding the recurrence rate and the neurological outcomes through a long follow‐up.

We here report two cases of hydrocephalus caused by FVOO and treated with ETV. Second, we systematically reviewed the medical literature for other published cases to draw inferences about the efficacy of ETV in treating FVOO. The recurrence rate was selected as the primary outcome parameter. Clinical neurological findings and mortality were defined as secondary outcome parameters.

## 2 METHODS

We report two cases of FVOO, who were diagnosed and underwent ETV in our center. In the second stage, a systematic review following the PRISMA statement^7^ was conducted after protocol registration in PROSPERO (CRD42021281474). MEDLINE, Embase, Scopus, and Web of Science were screened for relevant articles published before December 31, 2022. Two physicians (AM, AMA) independently reviewed potentially significant studies in a two‐stage procedure (title/abstract phase and then full‐text phase) using Covidence software.^8^ Studies were eligible for inclusion if they reported cases of FVOO treated initially with ETV. Occlusive hydrocephalus cases with Chiari malformation, Dandy‐Walker malformation, tuberous sclerosis, tumor, or space‐occupying lesions (i.e., secondary FVOO) and trapped fourth ventricle cases were excluded. AM and AMA extracted the data into an Excel spreadsheet independently. Articles with dissenting decisions were discussed between the reviewers until uniform assessment was achieved. We conducted a descriptive statistical analysis of presented and own cases (the latter ones were treated as a separate cohort). The primary outcome was the recurrence rate (a proportion), defined by the need for re‐ETV or other invasive treatment (Magendieplasty or VP shunt). Secondary outcomes included time to recurrence, improvement of symptoms, and mortality.

### 2.1 Statistical analysis

STATA software was used for statistical calculations. We used descriptive statistics to present the variables using mean and standard deviation (follow‐up) or median and interquartile range (age and time to recurrence) for continuous ones and percentage/absolute numbers for categorical (sex, etiology and recurrence). We calculated the 95% confidence interval (95% CI) of the primary outcome (recurrence rate) using an exact binomial distribution.

## 3 RESULTS

### 3.1 Cases presentation

#### 3.1.1 Case 1

##### Clinical history (course from premature birth till first home discharge)

The first case was a girl, prematurely born in the 25th week of pregnancy. She experienced an intraventricular hemorrhage (IVH, grades 2–3) during her hospital stay, from which she recovered well. Regular sonographic checkups revealed, however, a significant expansion of the inner ventricular system, without a nonproportional increase of head circumference (from birth to the age of 3 months, she remained just under the 3rd percentile, before starting to cross this threshold; and reached a position slightly over the 25th percentile at the age of 3 years and stayed within it afterwards; as it is shown in Figure S1). At 4 months, the girl was discharged home following stabilization and a rather long observational period on the neonatology ward. Through this period, the girl was not introduced to our neurosurgical department, and afterwards, she stayed in follow‐up control through the pediatric department.

##### Clinical presentation (course from presentation to neurosurgical consultation)

**FIGURE 1:**
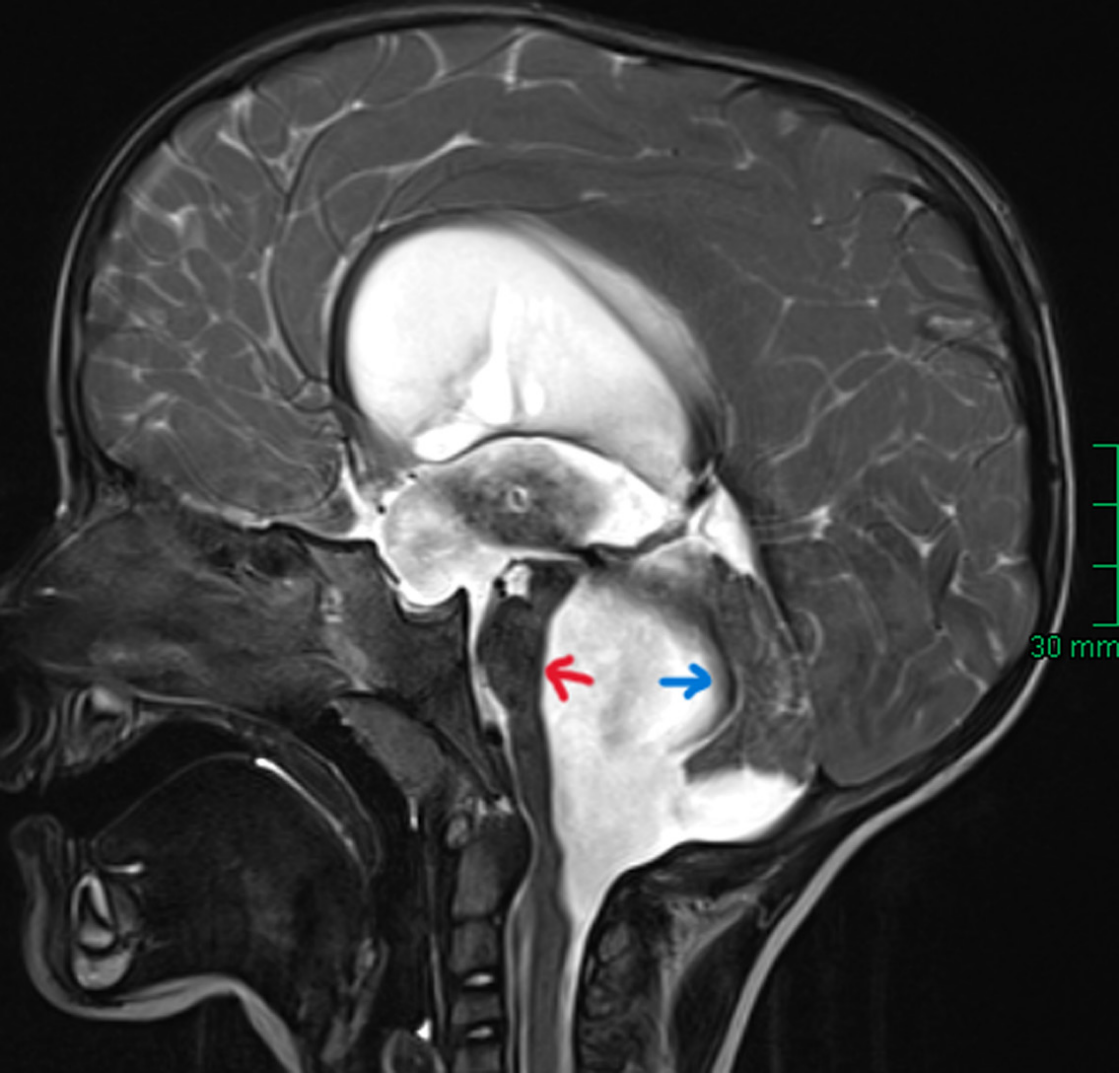
T2 sagittal MRI imaging of Case 1 showing hydrocephalus due to FVOO with a displacement of the brainstem (red arrow) and the cerebellum (blue arrow).

At the age of 3.5 years, the girl presented to the pediatric department with a noticeable deterioration in gait for several weeks. She had to hold on while walking and standing, and dysarthria developed. Following admittance, a cranial MRI was performed. It showed a progressive four‐compartment hydrocephalus internus (in comparison to earlier sonography studies). Compatible with FVOO, the brainstem was displaced rostrally and the cerebellum dorsally, indicating a severe compression of the posterior fossa structures (Figure 1).

##### Neurosurgical treatment (course from surgery till second home discharge)

**FIGURE 2:**
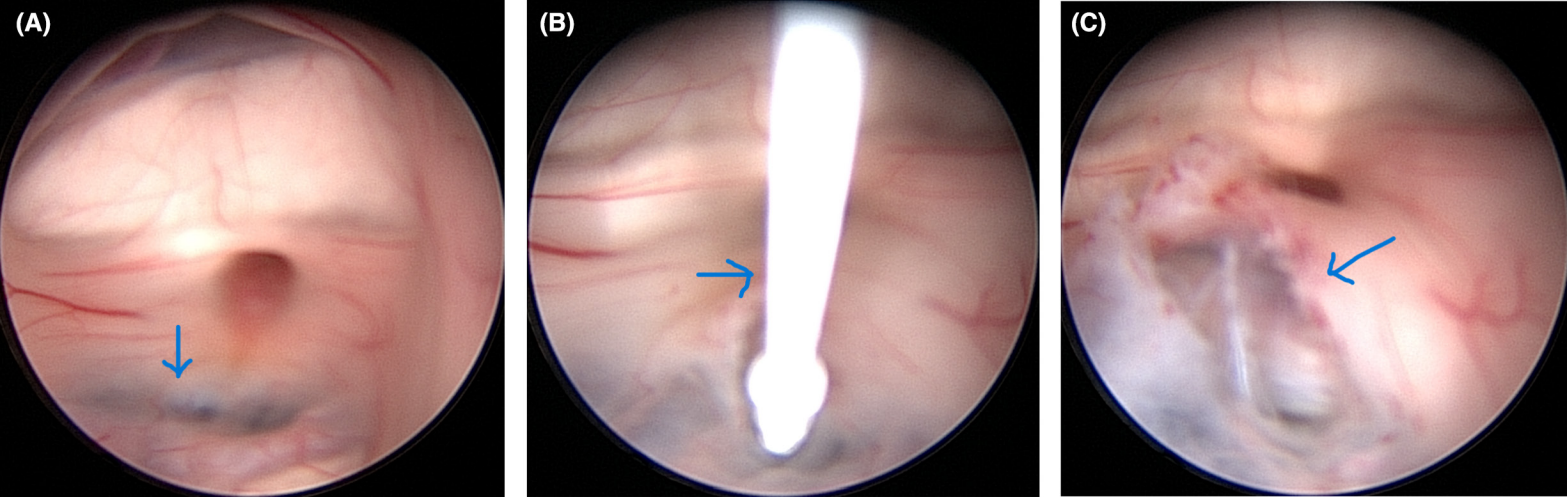
(A) Intraoperative endoscopic image showing the floor of the third ventricle (blue arrow). (B) Intraoperative endoscopic image showing the introduction of Fogarty catheter (blue arrow) into the third ventricle. (C) Intraoperative endoscopic image showing the stoma made at the floor of the third ventricle (blue arrow).

Neurosurgery was consulted, and ETV was indicated to avoid shunt dependency. During the procedure, a frontal burr‐hole was placed on the right side, and the rigid endoscope was introduced via the lateral ventricle and foramen of Monro to the third ventricle. The third ventricle floor was identified (Figure 2A), perforated by bipolar coagulation and then a Fogarty catheter was introduced (Figure 2B). Finally, a stoma was made by repeated inflation of the catheter's balloon. No intraoperative complications occurred, and the final intraoperative image showed a patent ventriculostomy (Figure 2C).

##### Postoperative findings (course after surgery)

Postoperatively, the patient was transferred to the neonatal intensive care unit for monitoring. Immediately after admission to the neonatal intensive care unit, there were recurring cerebral seizures, which could not be adequately interrupted by administering phenobarbital and diazepam. The persistent seizures were only stopped by additional therapy with lorazepam, clonazepam, and levetiracetam. From a neurosurgical perspective, the postoperative seizures are most likely explained by surgery‐related intracerebral pressure changes. Transcranial sonography revealed a consistent size of the third ventricle and lateral ventricles. Neurologically, the patient showed a gradual improvement. The wound healed without complications. One week after surgery, the early postoperative MRI revealed a patent ventriculostomy with a flow void, leaving the third ventricle into the external prepontine cisterns on flow‐sensitive TIRM sequences.

**FIGURE 3:**
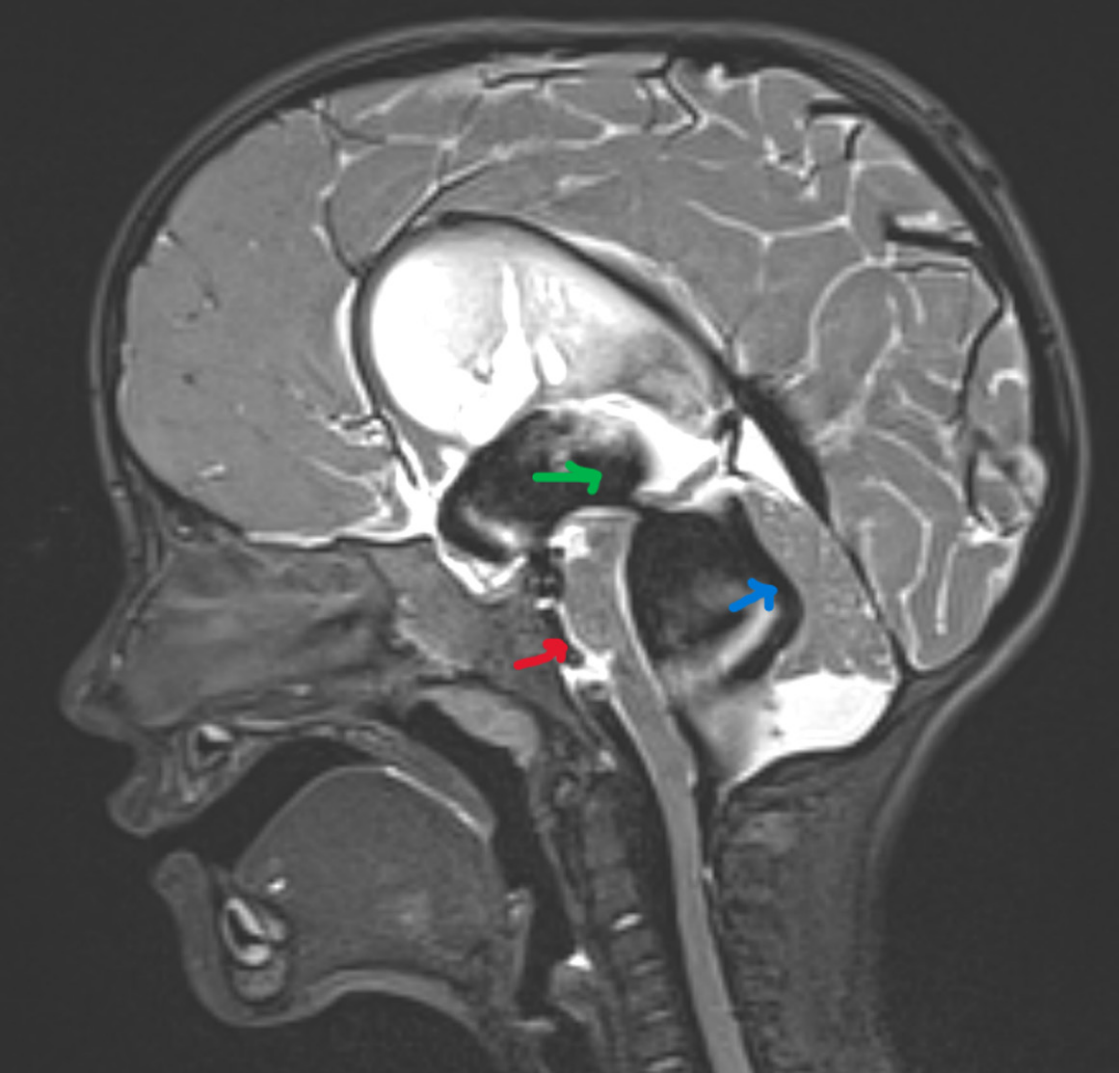
T2 sagittal MRI imaging of Case 1 shows a normal position of the brainstem (red arrow) and regression of the compression of the cerebellum (blue arrow) 1 week after ETV with flow void through the ventriculostomy and the aqueduct (green arrow).

Interestingly, a strong flow signal could be detected from the top of the fourth ventricle through the aqueduct into the third ventricle and prepontine cistern, proving an efficient retrograde flow of cerebrospinal fluid through the aqueduct (Figure 3). Ten days after admission, the patient was discharged home in good condition. Gait and speech disorders were slightly improved.

##### Follow‐up (course on year after surgery)

**FIGURE 4:**
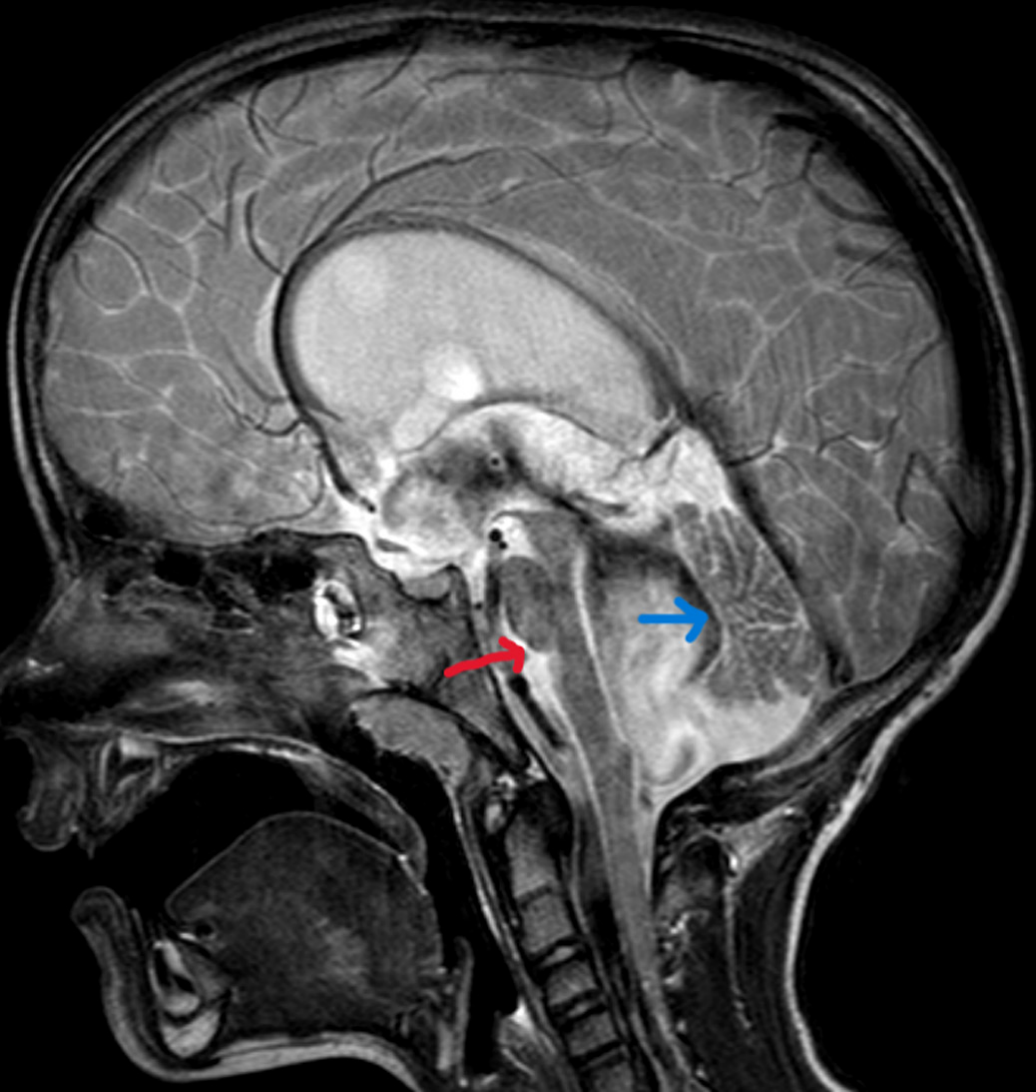
T2 sagittal MRI imaging of Case 1 shows a normal position of the brainstem (red arrow) and regression of the compression of the cerebellum (blue arrow) 1 year after ETV.

The patient underwent regular follow‐up examinations in the pediatric outpatient clinic, where she showed progressive improvement in neurological symptoms. At 1‐year follow‐up, there were no signs of ataxia or dysarthria, and an MRI showed a good regression of the compression of the brainstem and cerebellum with no displacement and still patent ventriculostomy and aqueduct (see Figure 4).

#### 3.1.2 Case 2

The second case was a premature boy who presented in 2013 at the age of 3 years. In the previous medical history, he had a grade 2 IVH, which had been treated conservatively. Neurosurgery was consulted because of progressive dysarthria and ataxia that developed 2 months before admission. Previously, the boy was able to stand up and walk normally. A cranial MRI showed a dilation of the four compartments of the ventricular system, indicating FVOO. The boy underwent ETV with no complications. The preoperative symptoms gradually improved, and no clinical or radiological recurrence was observed during the 1‐year follow‐up.

### 3.2 Literature review

**FIGURE 5:**
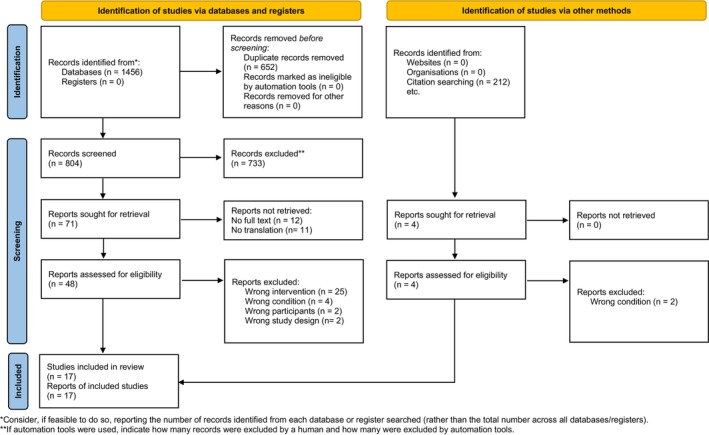
PRISMA 2020 flow diagram.

Searching the MEDLINE and Embase databases through the Ovid interface for the terms “fourth ventricle outflow obstruction”, “fourth ventricle outlet obstruction”, “FVOO”, or “magendie” or “Lushka” resulted in 1433 publication hits. Following screening and assessment for eligibility, 17 studies published between 2001 and 2021 were included in the review (Figure 5). Including our two cases, a total of 59 cases were found. Table 1 summarizes the demographic data and patient characteristics of the cases.

**TABLE 1:** characterization of all reported cases of fourth ventricle outlet obstruction (FVOO) treated with endoscopic third ventriculostomy (ETV), ordered ascendingly according to year of publication.

First author, Year of publication	*N*	Age in years/Sex	Symptoms	Cause of FVOO	Recurrence	Time in months	Revision	Follow‐up in months	Final Outcome
Carpentier, 2001^9^	1	58/F	Paroxysmal vertebrobasilar insufficiency/ICP symptoms	Idiopathic	No	–	–	36	+
Karachi, 2003^10^	3	21/F	Vertigo/ICP symptoms	Idiopathic	No	−	−	26	+
		53/F	Vertigo/ICP symptoms	Idiopathic	No	−	−	24	+
		68/M	Vertigo/ICP symptoms	Idiopathic	No	−	−	58	+
Monhanty, 2008^1^	20	−	−	Idiopathic in 12	Yes in 7 cases	1,5	VP shunt	50	?
Longatti, 2009^11^	7	53/M	Gait difficulties	−	Yes	144	Re‐ETV	144	+
		49/F	Gait difficulties	−	No	−		84	+
		70/M	Gait difficulties and incontinence	−	Yes	36	VP	72	+
		64/M	Gait difficulties and incontinence	−	−	−			?
		69/M	Gait difficulties and memory impairment	−	No	−		15	+
		69/M	Gait difficulties, memory impairment and incontinence	−	Yes	2	Re‐ETV	5	+
		58/M	Gait difficulties	−	−	−		2	+
Kawaguchi, 2009^12^	1	55/M	Syringomyelia syndrome	−	−	−	−	−	?
Dinçer, 2009^13^	2	3/F	Vomiting	Idiopathic	No	–	–	36	+
		1,5/F	Vomiting	Bleeding	No	–	–		
Oertel, 2010^14^	3	0,1/F	Increased head circumference	Bleeding	No	–	–	3	+
		0,5/F	Retardation, increased head circumference	Bleeding	Yes	?	VP	?	+
		28/F	Cephalgia, gait difficulties, vomitus	Infection	No	–	–	49	−
Roth, 2011^15^	1	0,6/?	Increasing head circumference percentiles	Idiopathic	No	−	−	26	+
Tabakow, 2013^16^	3	69/M	ICP symptoms	Idiopathic	No	–	–	5	†
		5/M	ICP symptoms	Bleeding	No	–	–	6	+
		24/F	ICP symptoms	Idiopathic	Yes	0,2	VP	1	?
Matsumura, 2014^17^	1	59/F	Ataxia, diplopia, and vomiting	Idiopathic	No	–	–	?	?
Hashimoto, 2014^18^	1	1,7/M	SIADH	Idiopathic	Yes	16	Re‐ETV	?	+
Ishi, 2015^2^	1	3/M	Headache and vomiting	Idiopathic	Yes	12	Re‐ETV	20	+
Spennato, 2019^19^	1	5/M	Headache, vomiting, drowsiness, parinaud syndrome, and left facial palsy	Idiopathic	No	–	–	60	+
Panero, 2019^20^	1	41/F	Headache, imbalance, and occasionally nausea and vomiting	Idiopathic	No	–	–	6	+
Chowdhury, 2020^21^	1	0,3/M	Increasing head size, feeding difficulty, respiratory distress, and tense fontanel	Idiopathic	Yes	1	Re‐ETV	14	+
El Damaty, 2020^3^	5	0,7/?			No				
		0,7/?			No				
		1,5/?	?	?	No	?	?	?	?
		?/?			Yes		VP		
		?/?			Yes		VP		
Krejčí, 2021^6^	5	22/F	Headaches, vertigo, gait disturbance, diplopia	Idiopathic	No	–	–	132	+
		74/F	Headaches, Hakim triad, vomiting	Idiopathic	No	–	–	84	+
		64/F	Hakim triad	Idiopathic	Yes	?	VP	24	+
		22/F	Headaches, vomiting, gait disturbance	Idiopathic	No	–	–	48	+
		39/F	Headaches, vomiting, vertigo, and papilledema	Idiopathic	Yes	1,5	Re‐ETV	7	+
Present study	2	3,5/F 4/F	Ataxia, dyspraxia, and dysarthria	Bleeding	No	−	−	3	+

Abbreviations: –, deterioration; ?, not mentioned; +, improvement; F, female; ICP, intracranial pressure; M, male; *N*, number of cases; SIADH, syndrome of inappropriate antidiuretic hormone secretion; VP Shunt, ventriculoperitoneal shunt.

^†^ Death.

The median age was 26 years, with an interquartile range from 2.4 to 59 years. 56% of cases were females. Most cases presented because of gait disturbance or signs of increased intracranial pressure with vomiting and headache. The most frequently reported cause of FVOO was idiopathic (19 cases), followed by bleeding (6 cases) and infection (1 case). No specific cause was reported in all other cases (33 cases).

The reported recurrence rate was 32% (19 of 59 patients, 95% CI from 21% to 46%). The median time to recurrence was 2 months (IQR from 1.25 to 26). Most recurrent cases (13 patients, resp. 68%) were treated by placement of a VP shunt, and re‐ETV was performed in the remaining six cases (32%). At the follow‐up (41 ± 29 months), only one case died, and one deteriorated clinically; all others improved.

## 4 DISCUSSION

In this study, we reviewed the reported cases of FVOO initially treated by ETV. The success rate concerning the primary endpoint was 68%. The study documents the efficacy of this minimally invasive procedure in treating this rare condition. FVOO differs from other types of obstructive hydrocephalus clinically by the striking cerebellar symptoms and radiologically by the massive dilatation of the fourth ventricle,^1^, ^9^ which is reflected by the dominance of gait disturbances and signs of increased intracranial pressure, such as vomiting and headache as the most common presenting symptoms, and macrocephaly in young children. It has, however, to be differentiated from isolated fourth ventricles, which might require further procedures, such as aqueductoplasty and stenting.^22^

Pathophysiologically, this condition is characterized by an obstruction of the outlet of the fourth ventricle composed of both Lushka (on the lateral sides) and the Magendie (median) foramina. At the same time, communication through the aqueduct remains open. This obstruction leads to a four‐compartment hydrocephalus internus. The underlying cause of the obstruction is mostly unknown (idiopathic)^1^, ^11^, ^12^; however, some patients who suffered previously from meningitis or intraventricular bleeding could later develop FVOO,^14^, ^16^ as was the case in our two pediatric patients.

Our systematic review included only “primary FVOO” cases without concurrent Chiari malformation, Dandy‐Walker malformation, tuberous sclerosis, tumors, aqueduct stenosis, or space‐occupying lesions. These conditions could result in “secondary FVOO”, and their treatment differs substantially from the treatment of primary FVOO, and including these secondary FVOO cases would introduce confounding in the interpretation of treatment outcomes.

Although it is more likely to encounter primary FVOO in the pediatric population, Longatti 2009^11^ and Krejčí 2021^6^ reported many adult cases, which found its expression in the demographic data of our systematic review, where FVOO was found to typically affect individuals in the first or sixth decades of life, with a median age of 26 years.

Historically, primary FVOO has been managed with suboccipital craniotomy with magendieplasty or VP shunt placement.^1^, ^6^ However, these approaches are invasive and associated with significant postoperative complications. In the case of VP shunt, they further require implants and cause shunt dependency. In recent years, ETV has emerged as a less invasive alternative for treating hydrocephalus in general and FVOO. Nevertheless, there is a scarcity of reported cases and limited evidence supporting the efficacy of ETV for this specific condition, particularly in terms of neurological improvement and recurrence rates. Theoretically, flow reversal in the aqueduct might eventually cause occlusion and development of a trapped fourth ventricle. Our analysis of reported cases showed that ETV successfully treated primary FVOO in approximately two‐thirds. Recurrent hydrocephalus occurred mostly within the first 2 months of treatment, and all could be treated by VP shunt placement or re‐ETV. Only one of the reported cases resulted in neurological deterioration, caused by the development of an isolated fourth ventricle 49 months following ETV.^14^ The only fatal case reported was due to cardiorespiratory failure unrelated to the neurological condition.^16^

Our findings contribute to the limited body of knowledge regarding this rare condition and its optimal treatment. They suggest that ETV is a safe and effective treatment modality, with a moderate recurrence rate and no surgery‐related severe adverse events in the reported cohort. ETV offers the advantage of being a minimally invasive procedure, avoiding the need for shunt placement and potentially reducing the risk of shunt‐related complications. However, careful patient selection and close follow‐up are crucial to monitor for potential recurrence and ensure timely intervention.

Limitations of the presented study are the limited sample size and the partly incomplete datasets in the reviewed literature because of the rareness of the reported condition and the retrospective nature of reports.

## 5 CONCLUSIONS

In conclusion, our study provides evidence supporting the efficacy of ETV in treating primary FVOO, demonstrating favorable outcomes in most cases. ETV should be considered a viable and less invasive alternative to traditional surgical approaches such as VP shunt or suboccipital craniotomy.

Further research with larger sample sizes and prospective studies is needed to validate these findings and establish more accurate recurrence rates and long‐term outcomes.

## AUTHOR CONTRIBUTIONS

**Ali Mulhem:** Conceptualization; data curation; formal analysis; investigation; methodology; writing – original draft. **Abdul Masih Alsulaiman:** Data curation; methodology; writing – original draft. **Stefanie Hammersen:** Supervision; writing – review and editing. **Sven Kantelhardt:** Supervision; writing – review and editing.

## FUNDING INFORMATION

No funding was received for this study.

## CONFLICT OF INTEREST STATEMENT

The authors declare no conflicts of interest.

## ETHICS STATEMENT

Ethical approval was waived in this retrospective study with anonymized data.

## CONSENT

Written patient consent, signed by the parents of the two children, has been obtained and documented in our institutional archive.

## Supporting information


Figure S1.

## Data Availability

All data and materials used in this study are available for sharing with the researchers. Please get in touch with the corresponding author.
