# Supplementary material for: The endoscopic treatment of fourth ventricle outlet obstruction: Report of two children and systematic review
Source: Clin Case Rep. 2023 Nov 28;11(12):e8234. doi: 10.1002/ccr3.8234 (PMC10683030; doi:10.1002/ccr3.8234)
Supplement: Supplementary file 1 — Figure S1. [file CCR3-11-e8234-s001.docx]

**Supplementary matrials**

Figure S1: Black line is the measurements of the head circumference of case 1 since born till the last follow-up (age of 4), x-axis represents the age in initially in weeks, and after the birth and through the follow-up in months. Y-axis represents the head circumference in cm, other lines are the percentile curves (3, 10, 25, 50, 75, 90, 97).


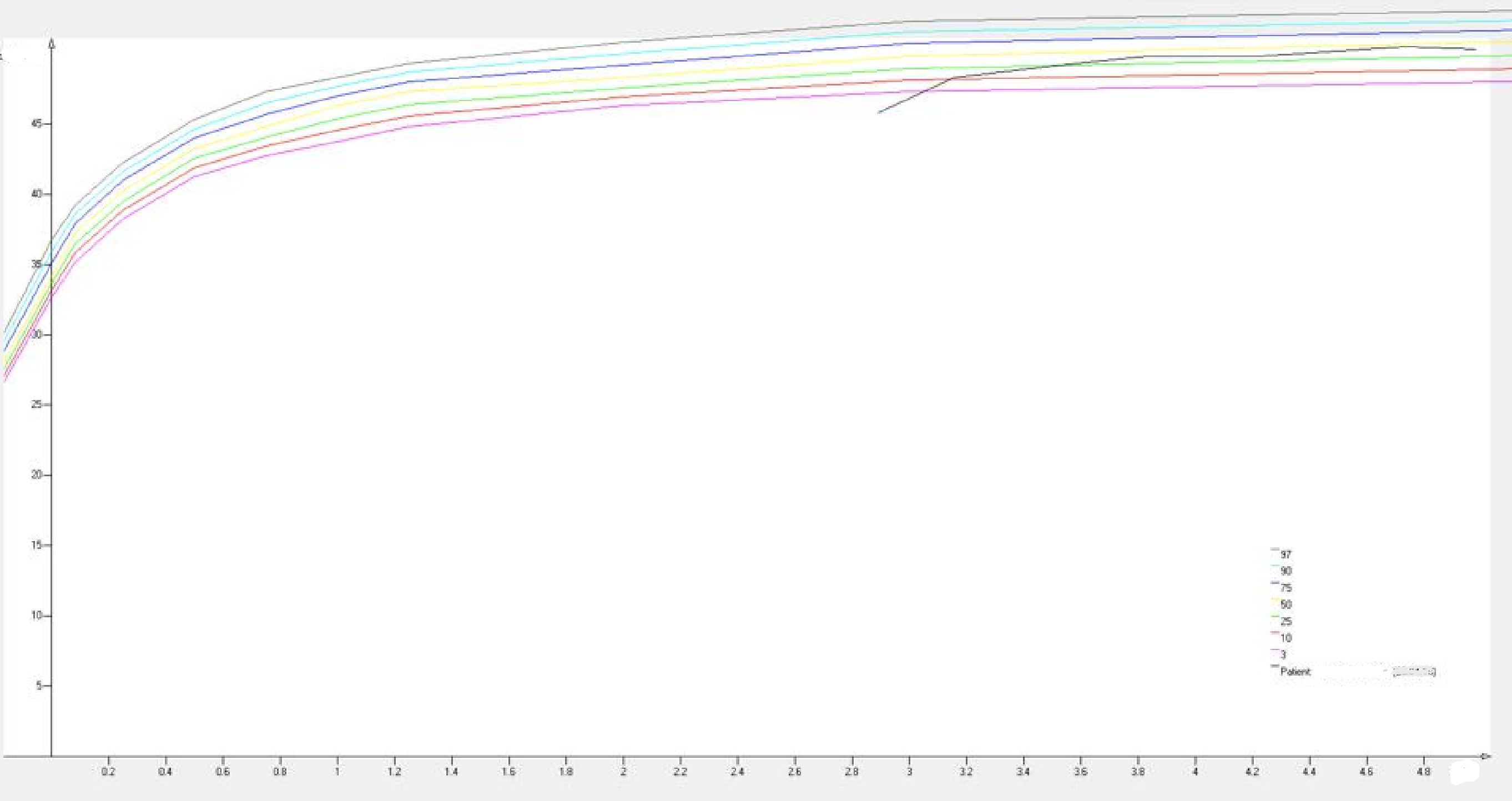

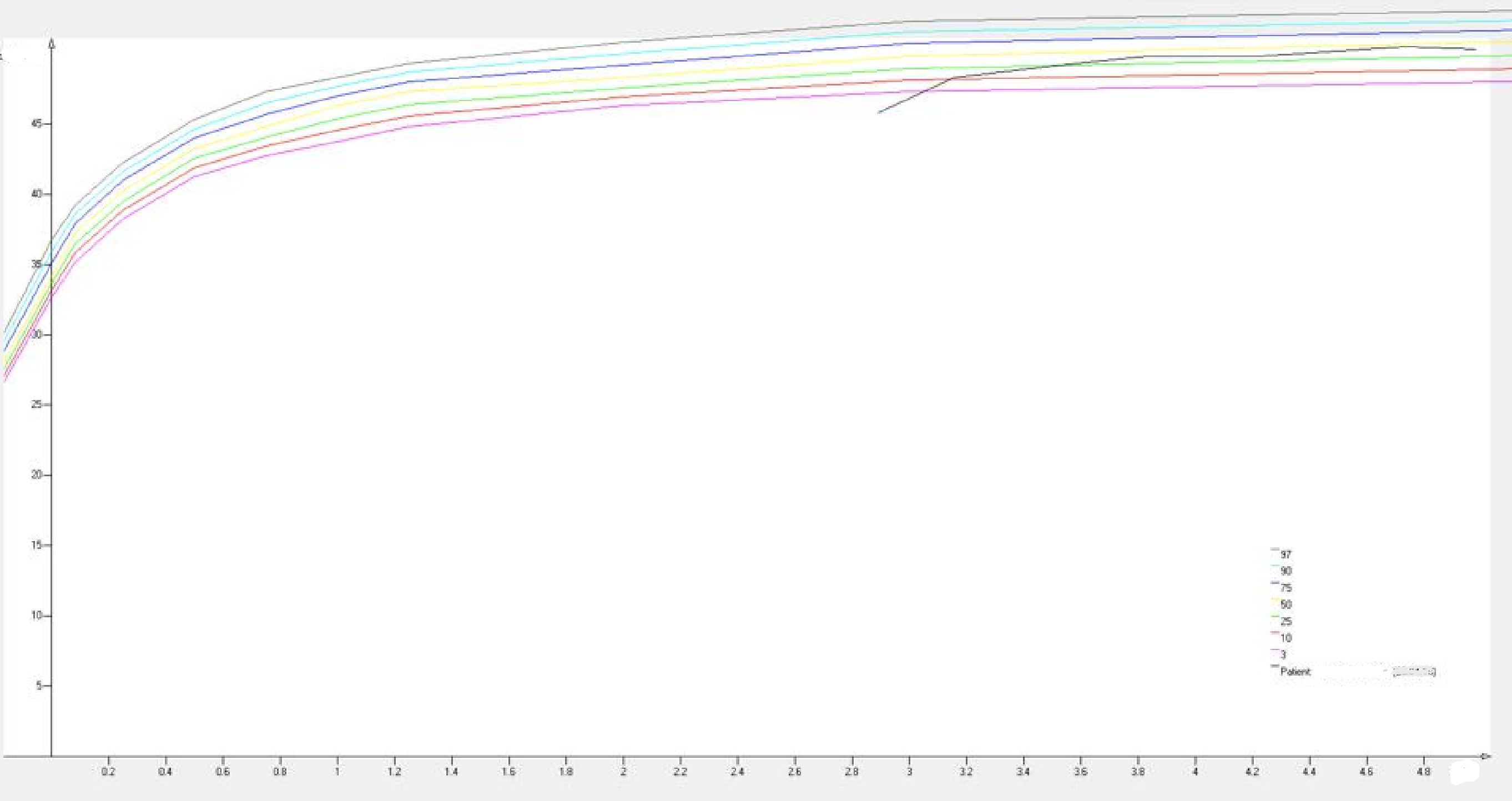


[cm]

45

40

35

30

25

[a]

97

90

75

50

25

10

3

patient

percentile
